# Autonomic Modulation for Cardiovascular Disease

**DOI:** 10.3389/fphys.2020.617459

**Published:** 2020-12-22

**Authors:** Joseph Hadaya, Jeffrey L. Ardell

**Affiliations:** ^1^University of California, Los Angeles (UCLA) Cardiac Arrhythmia Center, David Geffen School of Medicine, Los Angeles, CA, United States; ^2^UCLA Neurocardiology Research Program of Excellence, UCLA, Los Angeles, CA, United States; ^3^Molecular, Cellular, and Integrative Physiology Program, UCLA, Los Angeles, CA, United States

**Keywords:** vagus nerve, neuromodulation, neurocardiology, autonomic nervous system, heart failure, myocardial infaraction, arrhythmia, sympathectomy

## Abstract

Dysfunction of the autonomic nervous system has been implicated in the pathogenesis of cardiovascular disease, including congestive heart failure and cardiac arrhythmias. Despite advances in the medical and surgical management of these entities, progression of disease persists as does the risk for sudden cardiac death. With improved knowledge of the dynamic relationships between the nervous system and heart, neuromodulatory techniques such as cardiac sympathetic denervation and vagal nerve stimulation (VNS) have emerged as possible therapeutic approaches for the management of these disorders. In this review, we present the structure and function of the cardiac nervous system and the remodeling that occurs in disease states, emphasizing the concept of increased sympathoexcitation and reduced parasympathetic tone. We review preclinical evidence for vagal nerve stimulation, and early results of clinical trials in the setting of congestive heart failure. Vagal nerve stimulation, and other neuromodulatory techniques, may improve the management of cardiovascular disorders, and warrant further study.

## Introduction

The autonomic nervous system regulates all aspects of cardiac function, including chronotropy, inoptroy, dromotropy, and lusitropy (Fukuda et al., 2015). In the last decade, it has become increasingly evident that autonomic dysregulation plays a major role in the development and progression of major cardiovascular diseases, including myocardial infarction, heart failure, and sudden cardiac death (Fukuda et al., 2015; Ardell et al., 2016; Ardell and Armour, 2016; Shivkumar et al., 2016). While pharmacologic therapy targets cardiac disease through neurohormonal blockade, results have not been as promising as hoped (Florea and Cohn, 2014; McMurray et al., 2014; Shivkumar et al., 2016). This has led to increasing interest in neuromodulation as a new therapeutic approach, with therapies such as vagal nerve stimulation (VNS) and video-assisted thoracoscopic sympathectomy showing efficacy for the treatment of heart failure and refractory ventricular arrhythmias (Schwartz et al., 2004, 2008; Wilde et al., 2008; Bourke et al., 2010; Vaseghi et al., 2014; Sharma et al., 2020). Though these early studies are promising, a better understanding of the anatomy and function of the cardiac nervous system is imperative to developing and implementing precise neuromodulatory therapies.

**Figure 1:**
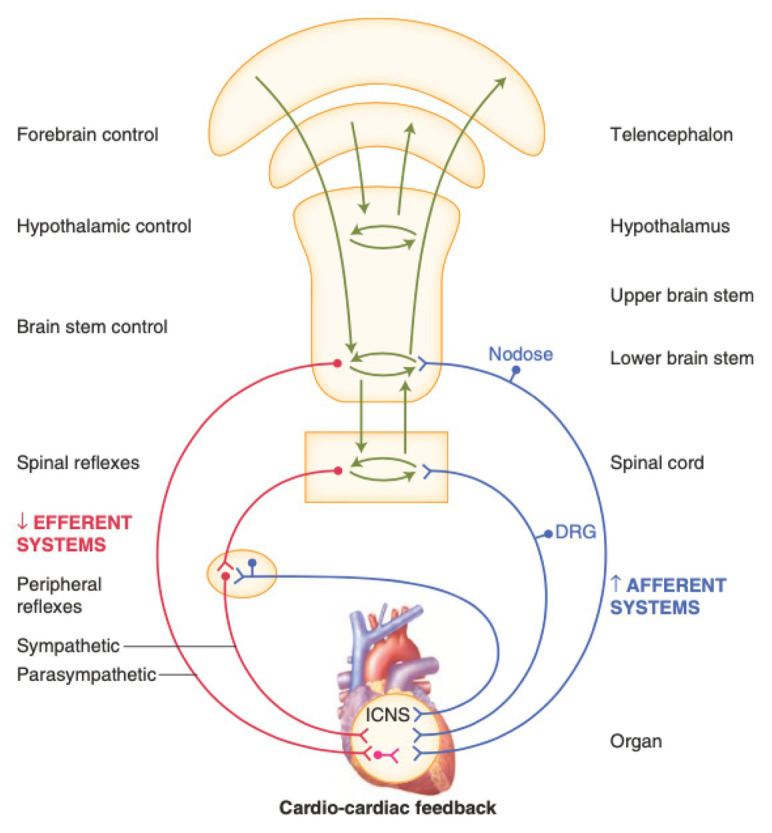
Neural regulation of cardiac function involves multiple nested feedback loops at the level of the heart, peripheral ganglia, and central nervous system. Afferent systems (blue) are mediated through the intrinsic cardiac nervous system (ICNS), dorsal root ganglia (DRG), and nodose ganglia. Efferent systems (red) involve sympathetic, parasympathetic, and local circuit neuron (LCNs). DRG, dorsal root ganglia. ICNS, intrinsic cardiac nervous system. Adapted from Jänig (2014) with permission.

The anatomy of the cardiac nervous system is complex and has been categorized into: (1) central components, (2) intrathoracic extracardiac components, and (3) intrinsic cardiac components (Figure 1; Fukuda et al., 2015; Ardell et al., 2016). The intrathoracic extracardiac system connects the central components to the intrinsic cardiac nervous system (ICNS), and is composed of parasympathetic and sympathetic systems that are traditionally thought to exert opposing action on cardiac electrical and mechanical function (Armour, 2008; Palma and Benarroch, 2014). The parasympathetic component acts through the vagus nerve and its intrathoracic branches, using acetylcholine, nitric oxide, and vasoactive intestinal peptide as neurotransmitters (Hill et al., 1995; Jänig, 2006). The sympathetic component originates in the intermediolateral cell columns of the spinal cord, projects *via* C7 to T6 rami to the superior cervical, middle cervical, stellate, or cervicothoracic ganglia, and acts though norepinephrine and neuropeptide Y (Norris et al., 1974, 1977; Jänig, 2006; Herring et al., 2012; Hoang et al., 2020). Afferent cardiac neurons, now known to be dispersed throughout the myocardium, act as mechanoreceptors and/or chemoreceptors, completing a cardiac neural circuit (Thompson et al., 2000; Armour and Ardell, 2004; Ardell et al., 2016). These afferent neurons have multimodal transduction capabilities, capable of transducing the presence of myocardial ischemia and (Fu and Longhurst, 2009) initiating reflex sympathetic activation in the setting of myocardial infarction (Malliani et al., 1969, 1973; Malliani and Montano, 2002; Billman, 2006). The ICNS is composed of clusters of ganglia referred to as ganglionated plexi distributed in epicardial fat pads (Armour, 2008). These ganglionated plexi contain a diverse population of neurons, including neurons that that receive parasympathetic and sympathetic input, neurons that receive afferent information directly from myocytes, and local interneurons (Armour, 2008; Ardell et al., 2016). Contrary to initial studies, recent evidence has shown that the ICNS is not simply a relay station for extrinsic projections to the heart, but instead functions with higher centers to modulate regional cardiac electrical and mechanical indices on a beat-to-beat basis (Cardinal et al., 2009). Moreover, the ICNS is a dynamic neural network, which remodels with disease states (Hardwick et al., 2008; Beaumont et al., 2015; Rajendran et al., 2016). For example, in the setting of myocardial infarction, our group has shown that processing of afferent and efferent neural signals in the ICNS is impaired, with overall decreased network connectivity, suggesting an inability of neurons to respond to local myocardial stimuli (Rajendran et al., 2016). Similar remodeling occurs within intrathoracic and primary sensory ganglia (Zucker et al., 2012; Wang et al., 2014; Ardell et al., 2016; Ajijola et al., 2017; Yoshie et al., 2020). Given the complexity of these systems, and remodeling that occurs in disease states, a better understanding of the function of each component is necessary to improve therapeutic outcomes and reduce off-target consequences.

In this review, we provide a brief foundation for understanding the structure of, and organization of the cardiac autonomic nervous system and highlight neural control of cardiovascular function, emphasizing the roles of the sympathetic, parasympathetic, and ICNSs. Based on this scientific foundation, an overview of current neuromodulatory therapies is presented, focusing on cardiac sympathetic denervation and VNS (Levy and Schwartz, 1994; Shivkumar et al., 2016; Hanna et al., 2018). We highlight preclinical studies of vagal nerve stimulation for structural heart disease and arrythmias. We conclude with a discussion on recently completed and in-process clinical trials of vagal nerve stimulation for heart disease, primarily congestive heart failure, driven by foundations established from preclinical studies.

## Overview of Structural and Functional Organization of the Cardiovascular Neuraxis

**Figure 2:**
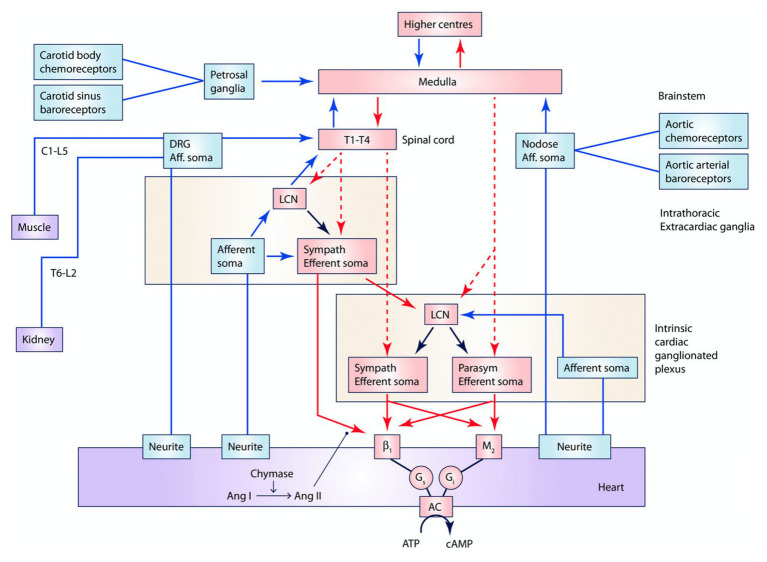
Interactions between central nervous system, peripheral ganglia, and the ICNS. Multiple interdependent feedback loops regulate regional cardiac mechanical and electrical function. At the level of the heart, sensory (blue) neurons provide input directly and indirectly through the ICNS, peripheral ganglia, and spinal cord, which are capable of engaging cardiocentric reflexes. Converging inputs result in activation of adrenergic or muscarinic receptors as well as receptors for peptide co-transmitters such as neuropeptide Y (not displayed). Preganglionic sympathetic and parasympathetic efferent fibers project (red, dashed) to the ICNS, including to LCNs, and postganglionic sympathetic fibers (red, solid) also directly to the myocardium. The ICNS acts as the final common pathway for cardiac control, though interactions occur at all levels. Input from chemoreceptors, baroreceptors, as well as neurohormonal factors such as angiotensin II and circulating epinephrine (not displayed), also modulate cardiac function. Ang I, angiotensin I. Ang II, angiotensin II. LCN, local circuit neuron. Adapted from Ardell et al. (2016) with permission.

Autonomic control of the cardiovascular system is mediated through afferent and efferent pathways and neural networks involving the brain stem, spinal cord, peripheral ganglia, and ICNS (Figures 1, 2; Ardell et al., 2016). Feedback systems between each of these components exist and mediate transduction of beat-to-beat information, allowing for maintenance of hemostasis and adaptation to stressors (Armour, 2008; Fukuda et al., 2015; Ardell et al., 2016). The cardiovascular neuraxis can be considered in three different levels: Level 1 is considered the heart with its own ICNS, which is comprised of cardiac ganglia that reside at the origin of the great vessels, and posteriorly along the atria and atrioventricular junction (Armour, 2008; Ardell et al., 2016; Shivkumar et al., 2016). Level 2 consists of intrathoracic components that regulate cardiac function including the middle cervical ganglia, cervicothoracic (stellate) ganglia, and the T2-T4 portions of the sympathetic chain (Kuntz and Morehouse, 1930; Armour and Janes, 1988; Ardell et al., 2016). Level 3 can be considered as the dorsal root and nodose ganglia, which mediates the majority of afferent neurotransmission, as well as spinal cord, brainstem, and higher centers (Malliani et al., 1975; Tjen et al., 1997; Armour and Kember, 2004; Jänig, 2006; Olshansky et al., 2008). Importantly, information is processed at multiple levels by interneurons, resulting in the interdependent network interactions between brainstem, spinal, intrathoracic, and cardio-cardiac reflexes (Cardinal et al., 2009; Ardell et al., 2015, 2016; Habecker et al., 2016). While initially thought to exert opposing effects on cardiac electrophysiologic and mechanical properties, interactions between the sympathetic and parasympathetic nervous systems mediate cooperative control and regulation of function (Linz et al., 2014; Fukuda et al., 2015; Ardell et al., 2016).

## Cardiac Sympathetic Innervation: In Health and Disease

Canonical organization of the sympathetic nervous system traditionally considers two sets of neuronal projections, defined by location of cell bodies and neurotransmitters mediating cell to cell communication. Preganglionic neurons with cell bodies in the intermediolateral cell column of the cervical and thoracic spinal cord (C7-T6) project to the superior, middle, cervicothoracic or stellate ganglia, and the remainder of paravertebral chain, with fibers running through ventral horns (Randall, 1994; Armour and Ardell, 2004; Jänig, 2006). Postganglionic sympathetic neurons project from these intrathoracic ganglia to atrial and ventricular myocardium, components of the conduction system, and coronary vasculature, traveling primarily as mixed cardiopulmonary nerves (Armour and Hopkins, 1981; Hopkins and Armour, 1984; Kawashima, 2005). Preganglionic neurons act through acetylcholine, binding to nicotinic acetylcholine receptors, whereas postganglionic neurons release norepinephrine. Sympathetic innervation of the heart has been well characterized using immunohistochemical studies targeting tyrosine hydroxylase, the rate-rate limiting enzyme in norepinephrine synthesis, across multiple animal models (Wharton et al., 1990; Kawano et al., 2003). Although canonical neurobiology primarily emphasizes the role of norepinephrine for postganglionic neurotransmission, several co-receptors such as neuropeptide Y and galanin have been reported to be released from postganglionic nerve terminals, especially during higher levels of neural activity (Lundberg et al., 1990; Hoover et al., 2009; Herring et al., 2012). These peptides may mediate additional efferent effects and have been implicated in various cardiovascular disorders including heart failure (Shanks and Herring, 2013; Ajijola et al., 2019). Further study of these co-transmitters may prove of value in development of novel therapeutics (Bohlender and Imboden, 2012).

In general, sympathetic activation mediates increases in chronotropy, inotropy, dromotropy, and lusitropy (Burwash et al., 1993; Fukuda et al., 2015). In more contemporary porcine studies, both right and left stellate stimulation reduced activation recovery intervals, an *in vivo* surrogate for action potential duration (Haws and Lux, 1990; Vaseghi et al., 2013). Notably, differential shortening of activation recovery intervals in different portions of the left ventricular (LV) by right versus left stellate stimulation was noted suggesting the presence of regional control of cardiac electrical function (Yanowitz et al., 1966; Vaseghi et al., 2012; Vaseghi et al., 2013). In the setting of chronic myocardial infarction, this regional regulation of cardiac electrical function is disrupted, with greater variation in repolarization and altered activation propagation (Ajijola et al., 2013).

In the setting of diseased states, such as myocardial infarction and heart failure, aberrant remodeling of the sympathetic nervous system occurs and is well described (Cao et al., 2000; Zhou et al., 2004; Ajijola et al., 2012, 2015). At the level of the myocardium, cardiac sympathetic nerve density, electrical excitability, and neurotransmitter content are altered in disease states (Cao et al., 2000; Gardner et al., 2015; Tapa et al., 2020). In a Langendorff-perfused mouse model of toxin-induced regional sympathetic hypoinnervation with no accompanying myocardial infarction, greater sensitivity to circulating catecholamines and blunted responses to sympathetic nerve stimulation were observed, demonstrating the direct proarrhythmogenic effects of sympathetic nerve heterogeneity (Tapa et al., 2020). Ischemia results in release of reactive oxygen species, adenosine, and other chemical mediators, that activate afferent neurons within the myocardium (Malliani et al., 1973; Dibner-Dunlap et al., 1993; Minisi and Thames, 1993; Huang et al., 1996). Reflexes at the level of the central nervous system, stellate ganglia, and possibly the ICNS, result in sympathoexcitation to mitigate in part the effects of ischemia on mechanical cardiac function (Minisi and Thames, 1991; Schwartz et al., 1992; Ardell et al., 2019). This reflex sympathoexcitation is associated with structural remodeling of portions of the ICNS as well as the stellate ganglia and an increased risk of ventricular fibrillation (Schwartz et al., 1976; Armour, 2004; Mill et al., 2011; Han et al., 2012). In porcine models of chronic ischemia, as well as humans with ischemic cardiomyopathy, histologic changes in stellate ganglia neurons have been observed resulting in inflammation, glial cell activation, and oxidative stress (Ajijola et al., 2012, 2015). Cholinergic transdifferentiation has been described following myocardial infarction, and, in animal models, co-release of acetylcholine with norepinephrine may be antiarrhythmic by reducing actional potential duration dispersion (Olivas et al., 2016; Wang et al., 2020).

From a clinical perspective, this excessive sympathoexcitation is targeted pharmacologically with beta blockers as well as inhibition of the renin-angiotensin-aldosterone system in the case of heart failure (Triposkiadis et al., 2009; Florea and Cohn, 2014; Fukuda et al., 2015). Neuromodulatory approaches to decrease sympathetic tone, such as thoracic epidural anesthesia, stellate ganglion blockade, cardiac sympathetic denervation, and renal denervation have been utilized clinically to manage these disease entities (Triposkiadis et al., 2009; Bourke et al., 2010; Schwartz, 2014; Fukuda et al., 2015; Shivkumar et al., 2016). In the research setting, another avenue considers application of electrical energy to reversibly block nerve conduction. For example, kilohertz frequency alternating current applied to the sympathetic chain mitigated sympathetic outflow to the heart, reducing electrophysiologic indices associated with sympathetic chain stimulation (Buckley et al., 2017). Similarly, in animals with chronic myocardial infarction, axonal block applied to high thoracic paravertebral chain using charge-balanced direct current produced reversible block of sympathetic nerve activity and reduced ventricular tachycardia/ventricular fibrillation potential (Chui et al., 2017). These technologies and treatment approaches warrant further study given an increasing interest of neuromodulation as a therapeutic opportunity.

## Cardiac Parasympathetic Innervation: In Health and Disease

Similar to the structural organization of the sympathetic nervous system, the parasympathetic nervous system is classically described as preganglionic and postganglionic projections (Jänig, 2006; Ardell et al., 2016). Preganglionic fibers, originating in the dorsal motor nucleus and nucleus ambiguus of the medulla project near to, or to, their target organ through the vagus nerve and intrathoracic cardiopulmonary branches (Standish et al., 1994; Hopkins et al., 1996). Presynaptic fibers synapse on postsynaptic cell bodies located near or on the target organ, with neurotransmission mediated through acetylcholine and nicotinic receptors (Jänig, 2006). In the setting of cardiac autonomic system, a significant portion of postganglionic cell bodies are located within ganglia of the ICNS, and then directly project to myocardium, acting through release of acetylcholine (Yuan et al., 1993; Armour et al., 1998; Pauza et al., 2014; Petraitiene et al., 2014). These groups of neurons have been well characterized using markers such as the choline acetyltransferase, and vesicular acetylcholine transporter (Hoover et al., 2004).

In addition to mediating parasympathetic efferent input to the heart and other viscera, the vagus nerve carries afferent information from intrathoracic and intraabdominal viscera to the central nervous system (Schultz and Ustinova, 1996; Schultz, 2001; Jänig, 2006). Of particular relevance to the cardiovascular system, bipolar neurons located in the superior and inferior (nodose) ganglia of the vagus nerve transmit sensory information from the myocardium to the nucleus tractus solitarus (Kalia and Mesulam, 1980; Janes et al., 1986). Vagal afferent fibers are predominantly unmyelinated and multimodal and with reference to bioelectric interventions are thought to be preferentially activated at lower levels of current (Yoo et al., 2013; Ardell et al., 2015). Given the prevalence of afferent fibers in the vagus nerve, with some studies reporting upwards of 80% of fibers as providing afferent input to the CNS, relevance of fiber type and activation is particularly important for the use of vagal nerve stimulation for therapeutic purposes (Janes et al., 1986; Prechtl and Powley, 1990; Hoover et al., 2008).

Vagal afferent fibers carry a wide variety of information to higher centers and include atrial stretch receptors, multimodal receptors within ventricles, pulmonary stretch receptors, and cardiopulmonary chemoreceptors (Paintal, 1953, 1963, 1973). Early studies, employing recordings of the vagus nerve and nodose ganglia, identified neuronal activity that was synchronous with the cardiac cycle, and affected by hemodynamic changes such as varied preload or afterload (Thoren, 1976; Berthoud and Neuhuber, 2000; Schultz, 2001; Schultz et al., 2015). In an *in vivo* study in guinea pigs, epicardial application of substances such as nitroprusside, calcitonin gene-related peptide, histamine, and bradykinin elicited changes in nodose afferent neuronal activity, suggesting that this population of afferent neurons transduce to a wide variety of neurotransmitters and chemicals at the level of the heart (Thompson et al., 2000).

In experimental models, efferent activation of the vagus nerve produces a bradycardia and slows conduction through the atrioventricular node (Armour et al., 1975; Ardell and Randall, 1986). However, at low levels of current, which primarily activates afferent pathways, a tachycardia is induced, which is abolished with transection of the vagus nerve (Ardell et al., 2015). While effects of vagal nerve stimulation on the sinus and atrioventricular node are well characterized, innervation of the ventricles by the parasympathetic system has been debated. Cholinergic neurons are present and innervate the ventricles as demonstrated by immunohistologic studies, with a greater density at the base than apex (Kawano et al., 2003; Hoover et al., 2004). Functional studies in porcine models demonstrate global prolongation of activation recovery intervals with right and left sided stimulation, with no clear regional regulation by the left versus right vagus (Yamakawa et al., 2014). This coordination across all areas of the heart likely reflects the interganglionic interconnections occurring within the ICNS (Ardell et al., 2016). In addition to electrophysiologic effects, vagal nerve stimulation reduces inotropy, further supporting the existence of functional innervation of the ventricle by the vagus (Yamakawa et al., 2014). With respect to vagal nerve stimulation, transection of afferent pathways rostral to the electrode interface markedly enhancing suppression of all cardiac functions, reflecting the dynamic interactions of afferent and efferent mediated responses whenever bioelectric interventions are imposed (Schwartz et al., 1973; Ardell et al., 2015, 2017).

Recently, selective modulation of neuronal populations has been made possible through optogenetic techniques that employ light-activated ion channels to stimulate or inhibit neurons (Zemelman et al., 2002; Boyden et al., 2005). For example, in a Langendorff-perused murine model, optogenetic stimulation of tyrosine-hydrolase expressing neurons on the epicardial surface of the right atrium and right ventricle resulted in greater contractile forces and heart rate, with shortening of optically-mapped action potentials (Wengrowski et al., 2015). A similar study in a murine Langendorff-perfused model targeting cardiac parasympathetic neurons expressing choline acetyltransferase demonstrated bradycardia and heart block with illumination of the junction of the superior vena cava and right atrium, which was blocked by administration of atropine (Moreno et al., 2019). Other non-electrical approaches for neural stimulation including designer receptors specifically activated by designer drugs (DREADDs) or receptor activated solely by a synthetic ligand (RASSL), a family of engineered proteins that respond only to synthetic ligands and allow for modulation of G protein coupled receptor signaling (Sternson and Roth, 2014). Using excitatory DREADDs in a rat model of heart failure, Garrott et al. (2017) activated a population of oxytocin and glutamate co-releasing neurons in the hypothalamus that stimulated cardiac vagal neurons, which mitigated myocyte hypertrophy, collagen deposition, and ventricular dysfunction. These novel approaches highlight pre-clinical advances that may allow for subselective evaluation of specific parasympathetic neuronal populations.

In the setting of myocardial injury and disease, structural and functional remodeling occurs at multiple levels of the cardiac nervous system (Ardell et al., 2016; Shivkumar et al., 2016; Shivkumar and Ardell, 2016). While myocardial injury generally results in a net increase in sympathoexcitation, central parasympathetic tone is reduced, driving an interest in restoring parasympathetic tone to treat disease (Olshansky et al., 2008; Ardell et al., 2016; Shivkumar et al., 2016). Using a porcine model, Vaseghi et al. (2017) reported minimal changes in acetylcholine levels in the apex, anterior wall, and lateral wall of the left ventricle in healthy animals compared to those with chronic myocardial infarction. Alterations in the basal activity and input of parasympathetic nervous within the ICNS were also evident in the setting of myocardial infarction (Vaseghi et al., 2017). Specifically, neurons normally activated by vagal stimulation were reduced in the setting of myocardial infarction, while those normally suppressed became more active. Inputs to these neurons significantly changed, with increased sympathetic input in the setting of myocardial infarction (Vaseghi et al., 2017). Changes in composition of the nodose ganglia has also been reported in the setting of chronic myocardial infarction, with increased neuron size as well as an increase in tyrosine hydroxylase positive and calcitonin gene-related peptide positive cells, with a decrease in neuronal nitric oxide synthase positive cells (Salavatian et al., 2017b). Overall, these findings suggest that intra and extra-cardiac parasympathetic remodeling occurs in the setting of myocardial infarction, with reduced parasympathetic input, but intact parasympathetic pathways (Vaseghi et al., 2017). As such, one therapeutic approach may be to target these intact pathways using vagal nerve stimulation.

**Figure 3:**
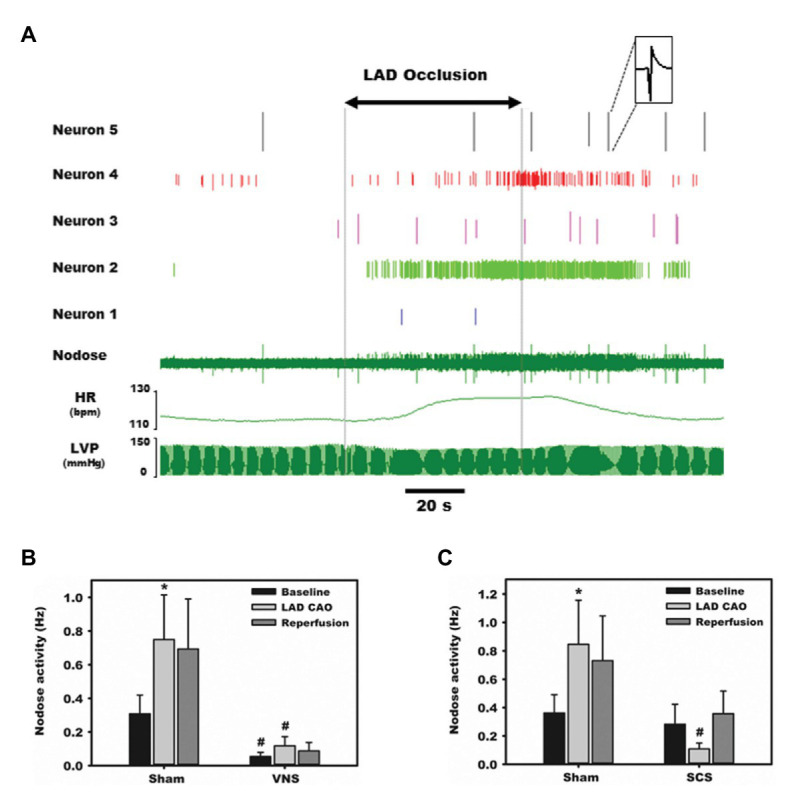
Afferent transduction of ischemia is modulated directly by parasympathetic input as well as remotely. Representative recordings from the nodose ganglia, one of the major parasympathetic afferent ganglia, during occlusion of the left anterior descending (LAD) coronary artery. **(A)** Activity from five neurons displays substantial heterogeneity in baseline firing frequency and responses to ischemia and reperfusion. **(B)** Normalized, summative nodose activity at baseline, with LAD artery occlusion, and reperfusion, with and without vagal nerve stimulation. In all settings, animals receiving vagal nerve stimulation (VNS) displayed marked reductions in nodose activity. **(C)** Spinal cord stimulation also reduces nodose neural activity in response to ischemia stress. Adapted from Salavatian et al. (2017a) with permission. ^*^p < 0.05 from baseline; ^#^*p* < 0.05 from sham.

Aberrant sensory information is a primary driver of acute autonomic responses to ischemic events and the progression of cardiac disease. Specific chemical ablation of cardiac transient receptor potential cation channel subfamily V member 1, TRPV1, receptors with epicardial resiniferatoxin mitigates progression of heart failure in preclinical models of ischemic heart disease (Wang et al., 2014). Targeted neuromodulation has the potential to impact sensory transduction in an on-demand and reversible fashion (Ardell et al., 2016; Salavatian et al., 2017a). Figure 3 shows the increase in nodose activity in response to transient occlusion of the left anterior descending (LAD) coronary artery. Such LAD occlusions were repeated in the presence of direct (cervical vagal, VNS) and remote (thoracic spinal cord, SCS) neuromodulation (Salavatian et al., 2017a). In both cases the nodose response to transient myocardial ischemia was mitigated (Salavatian et al., 2017a). Rather than being reflective of silent ischemia, we and others has proposed that pre-emptive neuromodulation alters the myocytes, likely *via* altered metabolism, and that this renders them stress-resistant (McGuirt et al., 1997; Southerland et al., 2007; Wang et al., 2014; Salavatian et al., 2016). As such the milieu is stabilized during transient ischemic events and sensory afferent responses and resultant evoked autonomic reflexes mitigated.

## The Intrinsic Cardiac Nervous System: Final Pathway for Cardiac Control

**Figure 4:**
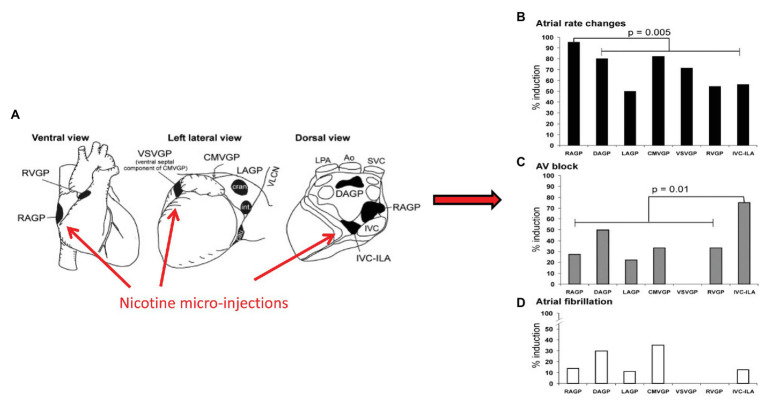
Aggregates of afferent, efferent, and interneurons located within epicardial fat pads comprise the ICNS. Although each ganglionated plexus has a preferred sphere of influence, substantial overlap exists. **(A)** Ventral, left lateral and dorsal views of the heart demonstrating common atrial and ventricular ganglionated plexi. Selected electrophysiologic effects of nicotine microinjections into major atrial and ventricular ganglionated plexi, **(B)** Heart rate, **(C)** Atrioventricular block, **(D)** atrial fibrillation. RVGP, right ventricular ganglionated plexus. RAGP, right atrial ganglionated plexus. VSVGP, ventral septal ventricular ganglionated plexus. CMVGP, cranial medial ventricular ganglionated plexus. LAGP, left atrial ganglionated plexus. DAGP, dorsal atrial ganglionated plexus. IVC-ILA, inferior vena cava-inferior atrial ganglionated plexus. Adapted from Cardinal et al. (2009) with permission.

The ICNS is a neural network comprised of afferent, efferent, and interneurons situated in fat pads distributed along the epicardial surface of the heart (Armour, 2008). Although the ICNS has been traditionally considered a relay station for efferent input to the heart, recent studies have identified a heterogenous population of neurons with varied putative functions (Armour et al., 1990; Butler et al., 1990; Ardell et al., 1991; Armour, 2008; Cardinal et al., 2009). These afferent neurons include multimodal and nociceptive afferent neurons, identified through both functional studies and immunohistochemical evaluation using antibodies against calcitonin gene-related peptide and substance P (Rysevaite et al., 2011a,b; Pauza et al., 2014; Ardell et al., 2016). As such, the ICNS has a role in afferent sensory transduction, local network processing afferent-efferent input and can be considered as the final common pathway for input to the heart (Armour, 2008; Ardell et al., 2016). While each aggregate of neurons has a preferential sphere of influence with regards to cardiac mechanical and electrical function, there is substantial overlap in areas of the heart regulated. For example, focal activation of all ganglionated plexi results in changes in heart rate, suggesting direct and indirect input from each GP to the primary pacemaker of the heart (Figure 4; Armour, 2008; Cardinal et al., 2009). Likewise, the nicotine micro-injections of most, but not all, ganglionated plexi can result in atrioventricular block or atrial fibrillation, and most have varied effects on ventricular mechanical function and electrophysiology (Armour, 2008; Cardinal et al., 2009). These data reflects the ICNS role in overall coordination of cardiac function, as a result of local circuit neuron (LCNs) mediating intra- and interganglionic interactions, shared efferent inputs, and convergent/divergent afferent inputs (Ardell et al., 2016).

**Figure 5:**
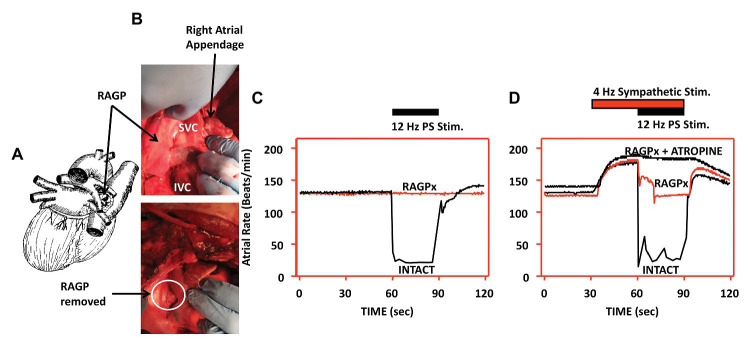
Interactions between sympathetic and parasympathetic system occur at end-effector as well as within the ICNS. Disruption of the RAGP alters neural control of heart rate. **(A)** Diagram depicting location of RAGP, posterior to the caval veins and anterior to right pulmonary artery and right superior pulmonary vein. **(B)** Photographs of RAGP before and after ablation. **(C)** Evoked bradycardia with 12 Hz parasympathetic stimulation in intact state, with loss of evoked bradycardia after RAGP ablation. **(D)** Combined sympathetic (4 Hz) and parasympathetic (12 Hz) stimulation in intact state, following RAGP ablation, and RAGP ablation with atropine administration. Adapted from McGuirt et al. (1997).

Interactions between the ganglionated plexi and higher centers ultimately modulate regional cardiac function, rather than each system acting on its own (Armour, 2008; Ardell et al., 2016). The right atrial ganglionated plexus (RAGP), located posterolateral to the superior and inferior vena cava, and anterior to the right superior pulmonary vein, is one of the major atrial ganglionated plexi and mediates significant parasympathetic input to the right atrium (Ali et al., 1990; Ardell et al., 1991; Cardinal et al., 2009). In animal models, ablation of the RAGP results in near complete loss of evoked bradycardia induced by vagal nerve stimulation (Figure 5; McGuirt et al., 1997). Sequential sympathetic and combined sympathetic-parasympathetic stimulation initially results in tachycardia followed by substantial bradycardia in the intact state (McGuirt et al., 1997). As shown in Figure 5, with ablation of the RAGP, the tachycardia induced by sympathetic stimulation is preserved but returned to baseline with the addition of VNS. This residual sympathetic-parasympathetic interaction is completely abolished when atropine is added (McGuirt et al., 1997). These observations suggest that interactions occur at both the level of end-organ as well as through the ICNS, underscoring the importance of such interactions in understanding regional regulation of function (McGuirt et al., 1997; Randall et al., 2003; Ardell et al., 2016).

The ICNS has received significant attention clinically due to its accessibility with cardiac interventions or cardiac surgery (Liu et al., 2013; Linz et al., 2014; Ripplinger et al., 2016; Shivkumar et al., 2016). Although dysfunction of the ICNS has been implicated in various cardiovascular diseases, our lack of knowledge regarding interactions among ganglionated plexi and higher centers has limited the advancement of therapeutics (Ardell et al., 2016; Shivkumar et al., 2016). For example, in 2009, Nakagawa et al. (2009) reported percutaneous ablation of five accessible atrial GP in 63 patients with paroxysmal atrial fibrillation, which resulted in a marked reduction of episodes of atrial fibrillation. Further follow-up of these patients, as well as other trials, showed a greater need for pacemaker implantation, likely due to disruption of neurons projecting to the sinoatrial node, and an inadvertent increased risk of ventricular arrhythmias in the setting of ischemia (Armour et al., 1997; Lo et al., 2013).

**Figure 6:**
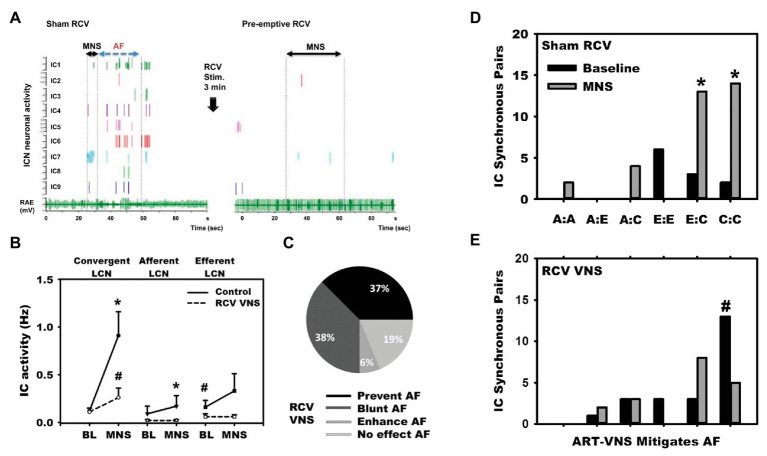
Vagal nerve stimulation targets LCNs within the ICNS to reduce arrhythmogenesis. **(A)** In response to mediastinal nerve stimulation, activity within the RAGP significantly increased and transient periods of AF were induced. **(B)** Increased neural activity during mediastinal nerve stimulation was primarily driven by increased activity of LCNs. **(C)** VNS mitigated neural activity changes in response to mediastinal nerve stimulation and prevented or blunted atrial arrhythmogenesis. **(D)** and **(E)** Intrinsic cardiac neurons were classified as afferent (A), efferent (E), or convergent (C). Mediastinal nerve stimulation resulted in increases in synchrony between efferent to convergent pairs and convergent to convergent pairs, while preemptive right cervical vagus nerve stimulation prevented such. Adapted from Salavatian et al. (2016). **(B)**: ^*^p < 0.05 from baseline; ^#^*p* < 0.05 from control (sham VNS). **(D)** and **(E)**: ^*^p < 0.05 from baseline; ^#^*p* < 0.01 sham to RCV VNS state.

**Figure 7:**
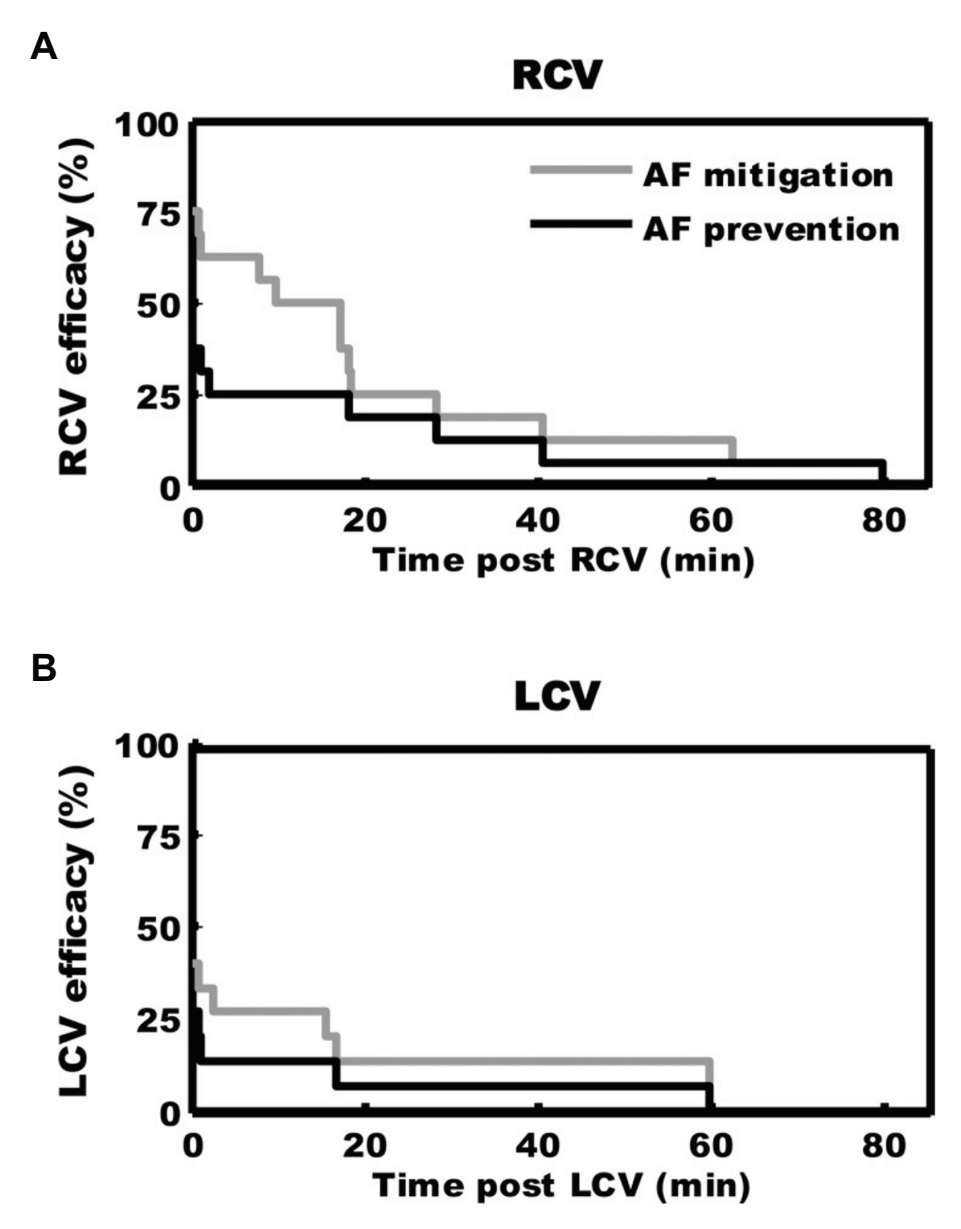
Kaplan-Meier curves demonstrating duration of antiarrhythmic effects of 3 min of preemptive cervical vagal nerve stimulation. After right **(A)** or left **(B)** sided vagal nerve stimulation, mediastinal nerve stimulation was less effective in inducing atrial fibrillation for approximately 30 min. Adapted from Salavatian et al. (2016).

Disruptions in autonomic inputs to the ICNS are arrhythmogenic. In a series of studies, our group developed a model for reproducible transient induction of atrial fibrillation (AF; Gibbons et al., 2012; Beaumont et al., 2013; Salavatian et al., 2016). Using this model, we recorded intrinsic cardiac neural and cardiac electrical activity in response to bursts of electrical activity delivered to cardiac mediastinal nerves during the atrial refractory period (Gibbons et al., 2012; Beaumont et al., 2013; Salavatian et al., 2016). Such bipolar stimulation reproducibly produces short periods of AF (~30 duration) with a latency of ~1 s (Figure 6). ICNS activity, derived from extracellular recording, can be functionally defined into afferent, efferent or convergent (both afferent and efferent) related. While burst stimulation of mediastinal nerves increases activity in all three functional classes of ICNS neurons, convergent neurons were the preferential target. As demonstrated in Figure 6, pre-emptive VNS reduces the evoked ICNS neural response to mediastinal nerve stimulation, with the primary target being the convergent neurons, and the atrial arrhythmia potential was reduced in 75% of stimulations. The VNS impact on ICNS network function is further exemplified by mitigating synchrony between efferent to convergent and convergent to convergent neurons (Figure 6, Panels D,E). Finally, VNS exhibits memory (Figure 7), specifically, 3 min of cervical VNS reduced the arrhythmia potential to neural imbalances produced by mediastinal nerve stimulation for approximately 30 min. This has important implications for design of stimulation protocols for VNS.

## VNS for Treatment of Cardiovascular Disease: Pre-Clinical Studies

**Figure 8:**
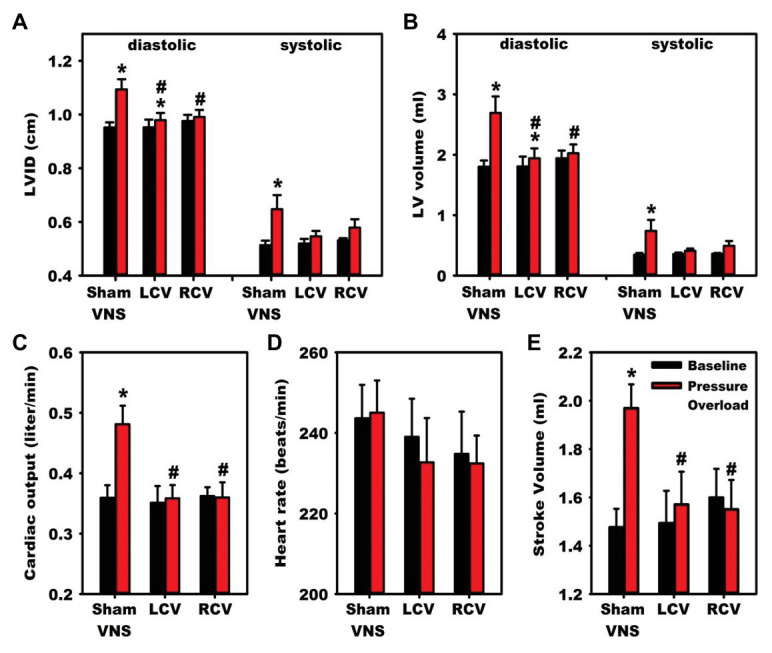
Vagal nerve stimulation mitigates heart failure and cardiac hypertrophy in a guinea pig model of chronic pressure-overload induced by aortic constriction. Compared to animals receiving sham vagal nerve stimulation, those receiving right or left sided VNS had improvements in indices of heart failure. Significant improvements were noted in diastolic indices of **(A)** left ventricular (LV) internal diameter and **(B)** LV volume. **(C)** Cardiac output and **(E)** stroke volume normalized to baseline with left and right vagal nerve stimulation, with no changes in (D) Heart Rate. LCV, left cervical vagus stimulation. RCV, right cervical vagus stimulation. VNS, vagus nerve stimulation. LV, left ventricular. LVID, Left ventricular internal diameter. Adapted from Beaumont et al. (2016). ^*^p < 0.05 from baseline; ^#^*p* < 0.05 to sham VNS.

Preclinical studies have provided substantial evidence supporting the utility of chronic VNS for the management of various cardiovascular disorders, with majority of studies focused on heart failure (Buckley et al., 2015; Schwartz et al., 2015). In a rat model of chronic heart failure induced by myocardial infarction through ligation of left coronary artery, 6 weeks of right vagal nerve stimulation ameliorated adverse ventricular remodeling and improved contractility (Li et al., 2004). Rats treated with VNS had reduced plasma norepinephrine and brain natriuretic peptide levels, and a 73% reduction in relative risk of death (Li et al., 2004). In a canine model of ventricular tachypacing-induced heart failure, concomitant right vagal nerve stimulation, at an intensity to reduce heart rate by 20 beats per minute, attenuated the development of heart failure (Zhang et al., 2009). Canine receiving concomitant VNS had improved LV ejection fraction and lower end-diastolic volumes, with greater heart rate variability and baroreflex sensitivity (Zhang et al., 2009). In a guinea pig model of chronic pressure overload designed to induce hypertension-mediated changes, chronic vagal nerve stimulation mitigated the development of cardiac hypertrophy and adverse neural remodeling (Figure 8; Beaumont et al., 2016). Compared to animals receiving sham VNS, those receiving right or left cervical vagal nerve stimulation had reduced pressure overload-induced remodeling, including a reduction in left ventricular internal diameter at diastole, left ventricular end diastolic volume, and cardiac output (Beaumont et al., 2016). At a cellular level, VNS consistently reduced pressure overload-induced myocyte hypertrophy and remodeling within intrinsic cardiac neurons (Beaumont et al., 2016). Taken together, these animal models suggest vagal nerve stimulation can modulate pathologic responses that result in heart failure, in part through improved autonomic balance, reduction of inflammation, alteration of energy utilization, and modulation of apoptotic pathways (Zhang et al., 2009; Zhao et al., 2013; Beaumont et al., 2016).

Preclinical studies have demonstrated putative anti-arrhythmic effects of vagal nerve stimulation in the setting of acute (Figures 6, 7) and chronic myocardial ischemia. In a study of acute coronary artery occlusion in anesthetized cats, animals who had previously undergone bilateral vagotomy or received atropine had less ventricular arrhythmias and death, suggesting that either efferent or afferent vagal tone may reduce ventricular fibrillation and death (Corr and Gillis, 1974). Billman et al. (1982) demonstrated that high vagal reflexes, assessed through baroreflex slopes, were associated with reduced vulnerability to ventricular fibrillation during acute coronary artery occlusion in canine with a history of healed myocardial infarction. Similarly, animals that were initially susceptible to ventricular fibrillation and underwent daily exercise had improved baroreflexes and reduced susceptibility to ventricular fibrillation, attributed to improved autonomic balance (Billman et al., 1984). In a canine model of healed anterior wall myocardial infarction, acute vagal nerve stimulation reduced the frequency of ventricular fibrillation in the setting of acute coronary artery occlusion and treadmill exercise among animals who were susceptible to ventricular fibrillation (Vanoli et al., 1991). In a similar study, canine who were resistant to ventricular fibrillation received atropine prior to coronary artery occlusion, resulting in either novel occurrence of ventricular arrhythmias or worsened degree of ventricular arrhythmias (De Ferrari et al., 1991). Taken together, these early studies support a putative benefit of vagal nerve stimulation and restoration of vagal tone in the setting of acute myocardial ischemia. In a rat model of acute coronary ischemia, concomitant vagal nerve stimulation reduced ventricular tachyarrythmias, with abrogation of this effect with atropine administration (Ando et al., 2005). Immunohistochemical studies showed that the vagal nerve stimulation mitigated the MI-induced loss of Connexin-43 at gap junctions, suggesting that vagal nerve stimulation may combat electrical instability that occurs with myocardial ischemia (Ando et al., 2005). In a contemporary, porcine study utilizing neuronal recordings from the ventral interventricular ganglionated plexus (VIVGP), a portion of the ICNS that innervates the ventricular myocardium, myocardial infarction resulted in increased sympathetic input to parasympathetic neurons in the VIVGP, with maintenance of parasympathetic neuronal networks. (Vaseghi et al., 2017) In this model, vagal nerve stimulation reduced the burden of ventricular arrhythmias and reduced heterogeneity of repolarization in the scar-border zone, an area known to initiate arrhythmias clinically (Rajendran et al., 2016). This study, and others, suggests that vagal nerve stimulation may, in part, act by stabilizing myocardial electrical activity, particularly at the scar-border zone.

**Figure 9:**
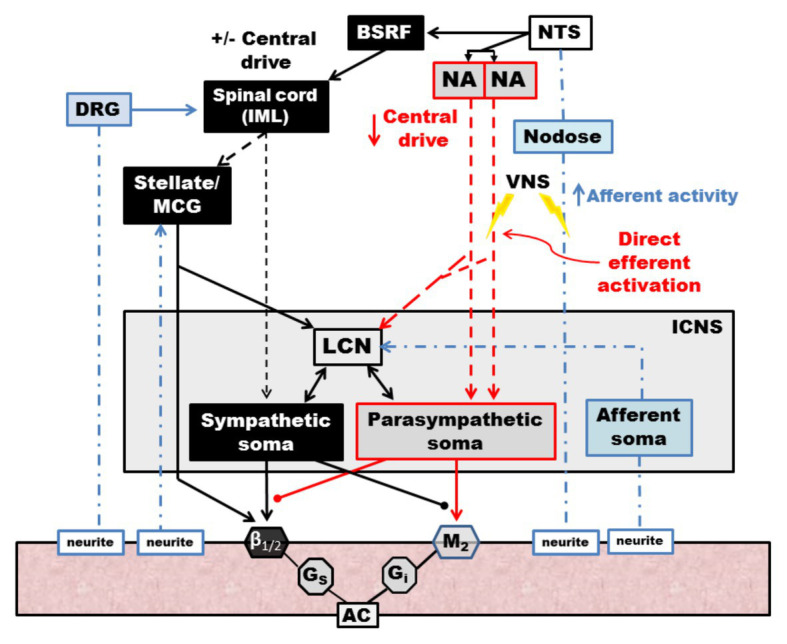
Schematic diagram of the effects of vagal nerve stimulation on the cardiac neuraxis. Direct efferent activation of the vagus nerve results in activation of parasympathetic postganglionic cells, located in the ICNS, which act on muscarinic receptors on the myocardium. LCNs within the ICN are activated, which modulate sympathetic reflexes with input from afferent soma, ultimately blunting reflexes within the ICNS. Afferent activation results in reduced central drive and modulates projections to spinal sympathetic networks. Sympathetic spinal reflexes are also blunted *via* inhibitory projections (not shown). NTS, nucleus tractus solitarus. NA, nucleus ambiguus. BSRF, brain stem reticular formation. VNS, vagal nerve stimulation. DRG, dorsal root ganglia. IML, intermediolateral nucleus. MCG, middle cervical ganglia. LCN, local circuit neuron. ICNS, intrinsic cardiac nervous system. AC, adenylyl cyclase. Adapted from Ardell et al. (2015).

Although the therapeutic molecular and cellular mechanisms underlying the therapeutic efficacy of vagal nerve stimulation are incompletely understood, mechanisms are thought to be mediated through both efferent activation of muscarinic receptors at the nerve-myocyte interface, as well as interaction with higher order systems and modulation of afferent-mediated reflexes (Figure 9; Ardell et al., 2016). At the level of the brainstem, afferent input is modified resulting in reduced central drive, as well as modulation of brainstem projections to spinal sympathetic networks (Ardell et al., 2016). At the level of the heart, activation of vagal efferent fibers results in acetylcholine release, activating M2 receptors on myocytes and conduction system cells, resulting in negative chronotropy, dromotropy, and, to a lesser degree, inotropy (Janig, 2016). VNS may, in part, alter the balance between energy demand and supply in the setting of pathologic states (Sabbah et al., 2011; De Ferrari, 2014). Coronary blood flow is modulated by vagal nerve stimulation with the majority of studies demonstrating parasympathetic coronary vasodilation although direct application of acetylcholine to vascular smooth muscle cells results in vasoconstriction (Winbury and Green, 1952; Schofield and Walker, 1953; Feigl, 1983). In a canine model employing bilateral vagotomies and bilateral stellectomy, efferent vagal nerve stimulation resulted in coronary vasodilation when controlling for heart rate and aortic pressure, which was partly mitigated by atropine administration (Feigl, 1969). At the level of myocytes as well as the ICNS, vagal nerve stimulation reduces sympathetic input, which may combat sympathoexcitation induced by disease states (Levy and Martin, 1979; Ardell et al., 2016; Vaseghi et al., 2017). On a molecular level, vagal nerve stimulation has been associated with reduced pro-inflammatory cytokine release and improved nitric oxide signaling (Tracey, 2007; Ruble et al., 2010; Calvillo et al., 2011; Sabbah, 2011). In addition, factors implicated in the progression of heart failure, such as norepinephrine and angiotensin are reduced with vagal nerve stimulation (Zhang et al., 2009).

## VNS for Treatment of Cardiovascular Disease: Clinical Trials

Congestive heart failure is a major public health problem in the United States, with greater than 6 million Americans affected, with an increasing prevalence in the last decade (Virani et al., 2020). Despite advances in medical therapies and interventional and surgical strategies for heart failure and comorbid conditions, mortality rates remain high as do the frequency of rehospitalizations for heart failure and associated costs (Benjamin et al., 2019).

Heart failure is a heterogenous disorder, resulting from various entities including myocardial ischemia and valvular diseases, which ultimately result in an inability to meet oxygen and metabolic demands (Fukuda et al., 2015; Gloschat et al., 2016). Homeostatic humoral regulatory mechanisms, such as the renin-angiotensin-aldosterone system, are activated in an attempt to maintain cardiac output and end-organ perfusion. Importantly, imbalance the sympathetic and parasympathetic nervous systems develop and contribute to the pathogenesis of heart failure, with, in general, withdrawal of parasympathetic tone and heightening of sympathetic tone (Olshansky et al., 2008; Triposkiadis et al., 2009; Fukuda et al., 2015; Gloschat et al., 2016). Consistent with this, the Autonomic Tone and Reflexes After Myocardial Infarction (ATRAMI) study reported an association between impaired vagal reflexes and mortality among 1,284 patients with a history of myocardial infarction, independent of left ventricular ejection fraction (La Rovere et al., 1998). The majority of pharmacologic therapy for heart failure targets changes in neurohormonal axis, including beta-blockers and angiotensin-converting enzyme (ACE) inhibitors (Triposkiadis et al., 2009). Guideline directed medical therapy for most patients with heart failure with reduced ejection includes beta-blockers and ACE inhibitors or aldosterone receptor antagonists (Yancy et al., 2013). Large scale randomized trials demonstrate an improvement in survival for ACE inhibitors and beta-blockers, particularly in those with a history of myocardial infarction or reduced ejection fraction (Pfeffer et al., 1992; Vantrimpont et al., 1997; Dargie, 2001; Verdecchia et al., 2009). Although these agents have been efficacious in the management of heart failure, progression of disease generally continues, leading to renewed interest in neuromodulatory therapies (Shivkumar et al., 2016).

Vagal nerve stimulation was initially developed for and clinically utilized for the management of depression and refractory epilepsy (Aaronson et al., 2013; Arle et al., 2016). Though the mechanisms of VNS for epilepsy are poorly characterized, reduction in seizure frequency and use of anti-epileptic drugs has been noted across trials (Ben-Menachem et al., 2015). The Food and Drug Administration has already approved implantable pulse generators and leads for safe and effective treatment of epilepsy, facilitating the adaptation of similar technology to alternate diseases (Ben-Menachem et al., 2015; Anand et al., 2019). Given the relevance of autonomic dysregulation in the development and progression of heart failure and arrhythmias, trials exploring the efficacy and safety of VNS for cardiovascular disease have received significant national attention since the initial report of its use for heart failure in 2008 (Schwartz et al., 2008; De Ferrari et al., 2011; Premchand et al., 2014; Gold et al., 2016; De Ferrari et al., 2017). Initial clinical trials of VNS, in combination with medical therapy, for CHF demonstrated mixed results, attributed to variation in study design, sidedness of stimulation, and vagal nerve stimulation protocols (Anand et al., 2020). Importantly, VNS protocols represent the total dose of “therapy” delivered to the patient as a function of current intensity, frequency, pulse width, and duty cycle (time on vs. off), and thus are hypothesized to have a substantial impact on efficacy of therapy, as well as side effects (Anand et al., 2020).

**Figure 10:**
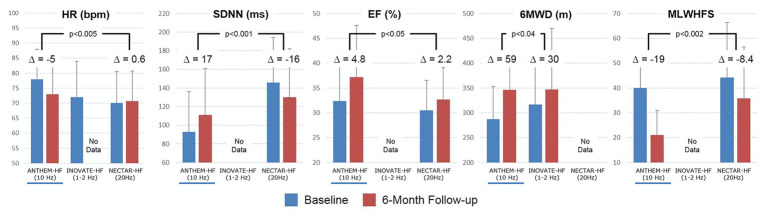
Clinical responses for the three major vagus nerve stimulation trials for HFrEF at baseline (blue) and 6-month follow up (red). Improvement for most parameters were noted for ANTHEM-HF, but unstudied or not met for INOVATE-HF or NECTAR-HF. Outcomes included heart rate (HR), standard deviation of NN intervals (SDNN, a heart rate variability time domain measure), ejection fraction, 6 minute walking distance test, and minnesota living with heart failure score. Adapted from Anand et al. (2020) with permission.

The Autonomic Neural Therapy to Enhance Myocardial Function in Heart Failure (ANTHEM-HF) evaluated the adjunct use of the Demipulse (LivaNova, Houston, TX) VNS system for left or right cervical vagal nerve stimulation in a cohort of patients with heart failure with reduced ejection fraction and New York Heart Association functional class II-III symptoms (Premchand et al., 2016; Dicarlo et al., 2018; Konstam et al., 2019; Sharma et al., 2020). Improvement in primary endpoints including left ventricular ejection fraction, heart rate variability, and 6-min walk tests, were noted, with follow-up at 1 year demonstrating persistent improvement (Figure 10; Premchand et al., 2016; Dicarlo et al., 2018; Konstam et al., 2019; Sharma et al., 2020). In the setting of heart failure with preserved ejection fraction (HFpEF), a more clinically challenging entity to treat, a similar VNS protocol by the same investigators is presently enrolling (Dicarlo et al., 2018). Of existing and completed trials for CHF, ANTHEM-HF is the only trial reporting positive findings for both safety and efficacy of VNS, which has been attributed to both study design and VNS stimulation protocols (Anand et al., 2020).

Two other major studies have explored the impact of VNS on cardiovascular outcomes in the setting of heart failure, with mixed results (Figure 10). The NEural Cardiac TherApy foR HF (NECTAR-HF) study randomized patients with heart failure with reduced ejection fraction (HFrEF) and left ventricular ejection fraction (LVEF) ≤ 35% to VNS therapy or sham (device without stimulation) with a different VNS protocol (intensity titrated to 20 Hz, 300 μs, proposed target current of 4 mA; Zannad et al., 2015). At 6-months, the majority of patients did not achieve the pre-specified stimulus intensity because of off-target effects and there was no significant difference in pre-specified outcomes of left ventricular end systolic diameter (Zannad et al., 2015). Importantly, NECTAR-HF targeted vagal nerve stimulation at a frequency of 20 Hz and a low current (Zannad et al., 2015). At these stimulation parameters, it is hypothesized that afferent fibers were preferentially activated, rather than efferent motor fibers, which may, in part, explain the lack of efficacy observed (De Ferrari et al., 2017; Anand et al., 2020).

The INcrease Of VAgal TonE in Heart Failure (INOVATE-HF) trial randomized 707 patients with HFrEF (LVEF ≤ 40%) with NYHA class III symptoms to medical therapy or medical therapy and VNS using an R-wave triggered pulse (Gold et al., 2016). Of note, this trial utilized a vagal nerve stimulation protocol that implemented a putative afferent block in addition to efferent activation (Gold et al., 2016). At a mean follow-up of 16 months, a composite primary endpoint of death, rehospitalization for CHF, or need for intravenous diuresis, was more frequent in the VNS group compared to control (Gold et al., 2016). About 6-min walk test and quality of life measures improved in the VNS group compared to control (Figure 10; Gold et al., 2016). The mixed findings in both of these studies may represent heterogenous cohorts and inadequate or inappropriately tailored VNS therapy delivery (Anand et al., 2020).

**Figure 11:**
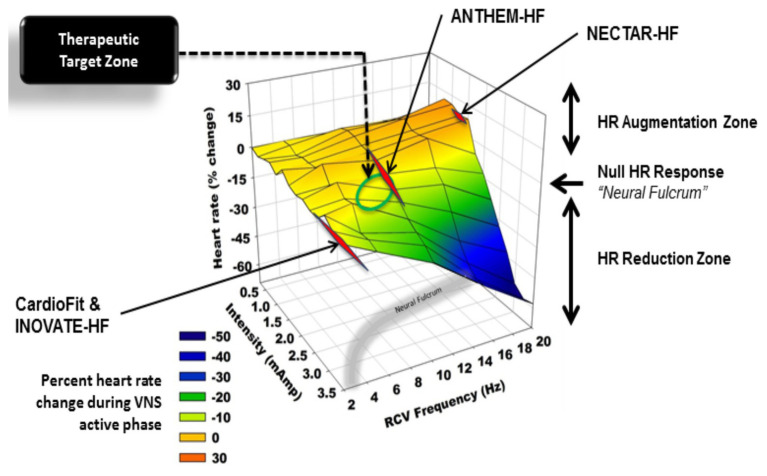
Heart rate responses to right cervical vagus nerve stimulation in a canine model at varying frequency and intensity (pulse width 500 μs). At higher frequencies and current, vagal nerve stimulation results in predominant activation of vagomotor efferent fibers, producing a bradycardia (blue). At higher frequencies and lower current, vagal nerve stimulation results in a relative tachycardia due to preferential activation of afferent fibers (orange, red). The area at which a null heart rate response is achieved is the neural fulcrum (yellow surface). Achieved stimulation parameters for three vagus nerve stimulation trials (ANTHEM-HF, NECTAR-HF, and INOVATE-HF) are plotted as a function of frequency and intensity. Adapted from Ardell et al. (2017) with permission.

Our prior work has described the concept of the neural fulcrum – an equilibrium point between activation of afferent and efferent vagus nerve pathways, resulting in a null heart response to stimulation with intact neural circuits (Ardell et al., 2017). Titration of vagal nerve stimulation parameters to reach the neural fulcrum over a period of time is a feasible approach to achieve therapeutic levels of VNS (Ardell et al., 2017; Anand et al., 2020). Likewise, adjustment of parameters during a training period, off-target effects such as cough, GI discomfort, and dyspnea, can be minimized (Ardell et al., 2017; Anand et al., 2020). Figure 11 represents a graphical display of a frequency and intensity relationship for VNS, with the neural fulcrum defined as the minimal heart rate change with VNS (yellow shaded region), overlaid with parameters from the three previously discussed trials of VNS for heart failure. At a given pulse width, in this case 500 μs, notes the inverse relationship between frequency and intensity to achieve a null and minimal bradycardia (1–3 beats per minute; Figure 11). Both NECTAR-HF and INOVATE-HF trials delivered VNS at parameters that were not effective in engaging all levels of the cardiac nervous system, while ANTHEM-HF operated at the fulcrum (Ardell et al., 2017; Anand et al., 2020). ANTHEM-HF is presently the only trial has shown a net long-term benefit to VNS for heart failure, which may be related to activating the appropriate balance of afferent and efferent fibers (Ardell et al., 2016, 2017; Anand et al., 2020). Although there were significant variation in clinical characteristics, disease burden, and comorbidities including arrhythmias, in these three trials, it is likely that the choice of VNS likely impacted clinical responses (Ardell et al., 2016, 2017; Anand et al., 2020). As future trials of VNS are designed for heart failure and other cardiovascular disorders, it is imperative that structure–function relationships of the nervous system be considered.

## Concluding Remarks and Future Directions

Multiple cardiovascular disorders including myocardial infarction and congestive heart failure result in excessive sympatoexcitation and a withdrawal of central parasympathetic tone (Ardell et al., 2016; Shivkumar et al., 2016). The majority of existing therapies for these disorders focus on blunting sympathetic drive to the heart, in the form of beta-adrenergic receptor blockade or inhibition of the renin-angiotensin-aldosterone axis (Shivkumar et al., 2016). Despite the widespread use of these medications, progression of disease occurs, creating a need for novel therapeutic approaches (Shivkumar et al., 2016). Therapies such as cardiac sympathetic denervation, stellate ganglion blockade, and renal artery denervation are being explored as avenues to reduce sympathetic tone associated with heart failure and ventricular arrhythmias (Shivkumar et al., 2016). In addition to the value of these therapies in the management of refractory ventricular arrhythmias, cardiac sympathetic denervation has also shown efficacy in the setting of inherited channelopathies including congenital long QT syndrome and catecholaminergic polymorphic ventricular tachycardia (Schwartz et al., 2004; Schwartz, 2014; De Ferrari et al., 2015). Vagal nerve stimulation has emerged as a promising therapeutic approach to combat the observed withdrawal of parasympathetic tone and to counteract the reflex mediated sympathoexcitation (Ardell et al., 2016; Shivkumar et al., 2016). A significant body of pre-clinical studies supports the value of vagal nerve stimulation for the management of heart failure or arrhythmias (Ardell et al., 2016; Shivkumar et al., 2016). Existing trials of vagal nerve stimulation have been confounded by substantial variation in inclusion criteria and inconsistent vagal nerve stimulation delivery (Ardell et al., 2016; Shivkumar et al., 2016; Anand et al., 2020). A mechanistic understanding of neural cardiac remodeling, where and how to target the cardiac neuraxis and follow-up with appropriate biomarkers is essential to achieve and maintain therapeutic efficacy. The goal for any of these therapies is to reduce sympathoexcitation, increase parasympathetic tone and/or blunt aberrant afferent processing. VNS achieves all three goals concurrently and remains an area of intense scientific and clinical interest.

## Author Contributions

JH and JLA contributed to writing, reviewing, and editing of the manuscript. Both the authors approved the submitted version.

### Conflict of Interest

University of California, Los Angeles has patents developed by JLA relating to cardiac neural diagnostics and therapeutics. JLA is a co-founder of NeuCures, Inc.

The remaining author declares that the research was conducted in the absence of any commercial or financial relationships that could be construed as a potential conflict of interest.
